# Blockchain-Based Solution for COVID-19 Digital Medical Passports and Immunity Certificates

**DOI:** 10.1109/ACCESS.2020.3043350

**Published:** 2020-12-08

**Authors:** Haya R. Hasan, Khaled Salah, Raja Jayaraman, Junaid Arshad, Ibrar Yaqoob, Mohammed Omar, Samer Ellahham

**Affiliations:** 1 Department of Electrical Engineering and Computer ScienceKhalifa University of Science and Technology 105955 Abu Dhabi 127788 United Arab Emirates; 2 Department of Industrial and Systems EngineeringKhalifa University of Science and Technology 105955 Abu Dhabi 127788 United Arab Emirates; 3 School of Computing and Digital TechnologyBirmingham City University 1725 Birmingham B4 7XG U.K.; 4 Heart and Vascular Institute, Cleveland Clinic Abu Dhabi Abu Dhabi United Arab Emirates

**Keywords:** COVID-19, blockchain, Ethereum, smart contracts, security, tracking, traceability, immunity certificates, digital medical passports

## Abstract

COVID-19 has emerged as a highly contagious disease which has caused a devastating impact across the world with a very large number of infections and deaths. Timely and accurate testing is paramount to an effective response to this pandemic as it helps identify infections and therefore mitigate (isolate/cure) them. In this paper, we investigate this challenge and contribute by presenting a blockchain-based solution that incorporates self-sovereign identity, re-encryption proxies, and decentralized storage, such as the interplanetary file systems (IPFS). Our solution implements digital medical passports (DMP) and immunity certificates for COVID-19 test-takers. We present smart contracts based on the Ethereum blockchain written and tested successfully to maintain a digital medical identity for test-takers that help in a prompt trusted response directly by the relevant medical authorities. We reduce the response time of the medical facilities, alleviate the spread of false information by using immutable trusted blockchain, and curb the spread of the disease through DMP. We present a detailed description of the system design, development, and evaluation (cost and security analysis) for the proposed solution. Since our code leverages the use of the on-chain events, the cost of our design is almost negligible. We have made our smart contract codes publicly available on Github.

## I. Introduction

Coronavirus-2019 (COVID-19) has had unprecedented impact on human life across the world. Being highly contagious, this disease has affected a significant proportion of the world population with a very large number of infections and deaths. With stringent countermeasures, such as lock-down adopted by governments across the world, COVID-19 has not only affected human health but has also caused a significant negative impact on the global economy. The symptoms of COVID-19 vary in severity across people and are similar to the influenza virus symptoms with fatigue, cough, high fever, and shortage of breath as the most common symptoms. However, not all people infected with the disease show symptoms. Some are known as silent carriers or silent spreaders, who do not show symptoms of the disease but carry and spread the disease to others [1]. Additionally, the disease has a very long incubation period which could last up to 14 days. During this period, the infected person can spread the virus without showing symptoms which makes effective and verifiable testing paramount to successful COVID-19 response.

As witnessed during the COVID-19 pandemic, the response strategy is evolved and implemented by the governments based on data related to infections collected by regional units. Such data relies on clinical diagnosis conducted by hospitals and other specialized facilities. However, the presence of multiple intermediaries in this process causes delays in reporting time which limits hospitals and testing centers from promptly responding in reporting infections [2]. Furthermore, layered structure in the reporting lines can also lead to discrepancies, thereby affecting the overall response strategies and their effectiveness to mitigate against the disease.

In this paper, we are focused on addressing the challenge of accurate and timely reporting of COVID-19 infections to aid response strategies against this disease. In this context, blockchain technology has introduced a new model of application development primarily based on the successful implementation of the data structure within the Bitcoin application [3]. The fundamental concept of the blockchain data structure is similar to a linked list, i.e., it is shared among all the nodes of the network where each node keeps its local copy of all the blocks (associated with the longest chain) starting from its genesis block [3], [4]. Recently, many real-world applications have been developed in diverse domains, such as the Internet of Things [5], e-Government [3], and e-document management [6]. These applications leverage benefits of blockchain technology because of its self-cryptographic validation structure among transactions (through hashes), and public availability of distributed ledger of transaction-records in a peer-to-peer network [3]. Creating a chain of blocks connected by cryptographic constructs (hashes) makes it very difficult to tamper the records, as it would cost the rework from the genesis to the latest transaction in blocks as illustrated by [3], [7].

We present an innovative blockchain-based solution to establish trust and eliminate fraud. In particular, our solution uses the programmable Ethereum smart contracts to execute function calls and generate events that notify participating entities about medical information, test updates, and requirements. Moreover, our design helps in curbing the spread of the COVID-19 virus through the use of on-chain digital medical passports and immunity certificates. Since the information spread on-chain is immutable, it can be trusted as it is from an affiliated source. Furthermore, in our proposed system, all announcements are made by trusted authorities that are affiliated by other higher authorities, such as the Ministry of Foreign Affairs (MoFA), Ministry of Health (MoH), and COVID-19 testing centers. We have also incorporated in our solution, self-sovereign identity as well as proxy re-encryption schemes along with distributed and decentralized storage systems.

### A. Related Work and Contributions

Herein, we review and summarize prominent works related to the COVID-19 pandemic especially considering blockchain applications to aid COVID-19 response.

Ting *et al.* [8] studied how different cutting-edge technologies can help in mitigating the spread of the COVID-19 disease. In particular, the authors highlight how the Internet of Things (IoT), big data, blockchain, and artificial intelligence (AI) can help in creating simulation models that predict the spread of the disease. Moreover, these technologies can aid in establishing a screening tool that can facilitate in terms of diagnosis and monitoring of the disease’s spread. With respect to real-life use-cases, the authors highlighted the use of blockchain to facilitate tracking deliveries of the medications to the patients’ doorsteps in China. The focus, in this effort, is on highlighting the potential usage of cutting-edge technologies to help mitigate COVID-19 spread. This study did not provide details on technical implementations. Similarly, Mashamba-Thompson and Crayton proposed blockchain and AI to achieve COVID-19 self-testing.

On the other hand, Torky and Hassanien [10] proposed an approach to use blockchain to automatically detect infected cases and estimate the infection risk of the COVID-19 in society. The authors utilized the decentralization property of blockchain to store the information and medical data of the confirmed COVID-19 cases. The detection of the infected cases depends on other technologies, such as an infection verifier subsystem and a mass-surveillance system.

Similarly, Nguyen *et al.* [11] proposed an approach to facilitate predicting the spread of the COVID-19 virus and other similar epidemics. They proposed using AI along with blockchain to process a large volume of medical data that has a complex pattern. The paper presented a blockchain-based approach to help with donation tracking and the healthcare supply chain. However, it does not provide technical details regarding the implementation.

Furthermore, Bansal *et al.* [12] demonstrated the use of blockchain in creating immunity certificates. The authors also proposed the use of immutable blockchain technology to avoid the spread of false reports and information. The proposed solution also attempts to address the challenge of privacy and anonymity of the test-takers. However, the authors have not included a design scheme or an implementation method to achieve the results of the proposal. Resiere *et al.* [13] proposed a blockchain-based method to revitalize the medical health system in the Caribbean. Therefore, they proposed the use of blockchain technology to achieve medical cooperation and collective scientific research to fight against the spread of COVID-19 infectious disease.

Finally, Kumar *et al.* [14] have developed an approach to improve the deep recognition of a deep learning model to recognize COVID-19 patients based on tomography (CT) slices. The authors compared their proposed approach with other deep learning models, such as VGG16, VGG19, DenseNet, AlexNet, MobileNet, ResNet, and Capsule Network. Their research uses blockchain as a means of sharing data while maintaining privacy.

In summary, although several avenues of using cutting-edge technologies, such as blockchain and IoT to facilitate COVID-19 response have been explored, the existing efforts do not present technical details except [14], where the authors show their deep learning model implementation details. Moreover, the aforementioned solutions do not show a method of mitigating the spread of the COVID-19 through the direct usage of blockchain technology. In most of the articles, blockchain is either proposed as a promising technology to curb the spread, help in stopping the spread of false information, or is accompanied by other technologies to propose a framework. None of the researches proposed and implemented a blockchain-based solution that can help track and trace COVID-19 test-takers through affiliated and authenticated immunity certificates and digital medical passports.

Our main contributions in this article can be summarized as follows:

• We propose a blockchain-based solution that offers tracking and tracing of COVID-19 test-takers. The proposed solution leverages the use of the immutable events and logs of the distributed blockchain ledger without relying on any on-chain storage.
• We manifest how self-sovereign identity (SSI) accompanied by our blockchain design is an effective and decentralized identity system.
• We use proxy re-encryption schemes to integrate our blockchain-based system with the InterPlanetary File System (IPFS) and securely store the patient and test-takers medical, identity, and travel information.
• We perform security and cost analysis of our solution to demonstrate its feasibility and reliability.
• We present the full implementation details, smart contract code,^1^ and testing details.^1^https://github.com/smartcontract694/covid19/tree/master

The rest of the paper is organized as follows. Section II presents the design details of the proposed blockchain-based solution followed by the implementation details in section III including the smart contracts and algorithms. Section IV presents the evaluation of the proposed system followed by section V which highlights the feasibility of our solution through a thorough cost and security analysis. Section VI concludes the paper.

## II. Proposed Blockchain-Based Solution

In this section, we present a detailed explanation of the design of the proposed blockchain system. Our solution employs Ethereum smart contracts and utilizes the immutable logs and trusted events. Our solution helps in tracing and tracking patients for their medical tests as well as travel history. It reduces the stress on employers, government facilities, social and academic services as well as transportation systems in creating an identity for its entities. It also helps in containing and mitigating the spread of the COVID-19 virus.

**FIGURE 1.**
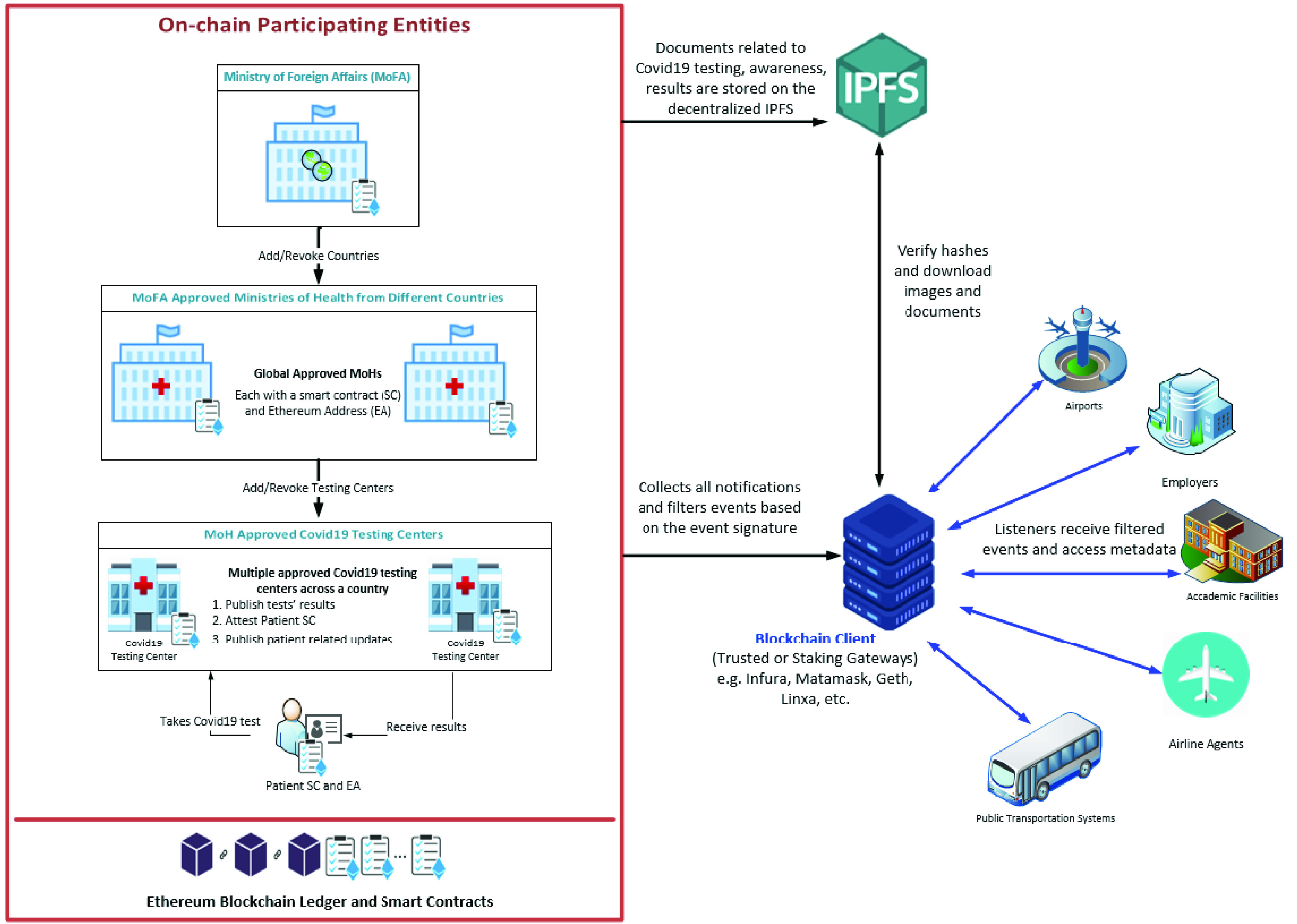
An overview of the proposed blockchain-based system for digital medical passports and immunity certificates.

Figure 1 shows the system diagram of the proposed blockchain-based solution. It presents the on-chain participating entities with different smart contracts, distributed storage, blockchain clients, and interested stakeholders. There are four main types of smart contracts used in our proposed solution i.e. i) the MoFA smart contract, ii) the MoH smart contract, iii) the COVID-19 Testing Center smart contract, and iv) the Patient smart contract. We present details of these smart contracts along with other sub-components of the system below.

### A. Digital Health Passports and Immunity Certificates

*Digital health passports* are a crucial mode of identification which can help mitigate the spread of contagious diseases. The patient smart contract is envisaged to address this objective. It is an immutable record that is authenticated by the MoH and the MoFA for international usage. The patient smart contract holds the IPFS hash of the vaccination and immunization records as well as the medical and travel history of an individual. In the context of the personally identifiable information used in this structure, the disclosure of the information is delegated to the owner of the information.

*Immunity certificates* are envisaged to verify that a person has developed relevant antibodies to mitigate against COVID-19 and is consequently not a a threat to (cannot infect) other people. We envisage this to have been achieved either through a past infection of COVID-19 or through vaccination (when Although we acknowledge the significance of this challenge, however, as our focus is on technological perspective of the challenge, we render the medical aspect of this challenge out of scope of this paper. Therefore, they can be exempted from physical and social restrictions as they are immune to the disease [12]. This information can also be part of the patient smart contract and it can also be announced using an immutable transaction by the COVID-19 Testing Center. The center can announce it after an antibody test and the time-frame the patient is immune would also be announced (depending upon the vaccine strength and relevant medical advice).

To date, the immunity period is still an inconclusive and debatable subject amongst scientists and researchers. However, this research is designed to accommodate the scientific progress done and is flexible to be changed based on its results. Between April and July, 2020, there were no published studies documenting or demonstrating protection from a secondary SARS-Cov-2 infection in patients with antibodies generated from the primary infection [15]. Yet, also there has not been any evidence of clinically and serologically proven independent secondary re-infection from an identical strain. However, in August, three studies have provided strong evidence of clinical immunity providing protection from re-infection by SARS-Cov-2 [16]–[18]. The immunity time frame is still being researched, and is currently known to still be valid in the short run. To address that, our design has taken care of it by including the time when an announcement is made about immunity certificates.

Since an immunity against the virus is a positive and promising news, the announcement can be done as a notification that is logged to the public. However, if the test-taker decides otherwise, there is another function that publishes updates and test results using IPFS hashes only. In our design, the COVID-19 Testing Centre smart contract can generate events to notify patients and test-takers about the medical updates. Those updates can be quarantine information or details about the medical tests that they have done. Therefore, being immune to the disease is an update that the Testing Center can communicate in an event to ensure that it is immutable. Private information or medical test results are disclosed using an IPFS hash only on-chain and the information on IPFS is further encrypted as detailed in the next sections.

### B. On-Chain Participating Entities: MoHs and MoFA

MoH and MoFA are important stakeholders within our solution. They represent authorities that ensure tests are legitimate and the results are all real. Every COVID-19 testing center must be affiliated with an MoH which is in turn affiliated with the MoFA. The MoH can add COVID-19 testing centers that meet their meet their requirements and can also revoke previously added COVID-19 testing center(s). All this is done by using the immutable events and transaction logs in the blockchain network.

Moreover, a MoFA can add MoHs and revoke them based on their requirements and regulations. The MoFA plays an important role in mitigating the spread of diseases across borders and around different territories. It only affiliates MoHs that meet their rules and regulations. This is also done through events which are communicated with the participating entities and interested audiences. COVID-19 Testing Centres that are affiliated can then conduct tests for registered test-takers and patients. Every individual’s biometric information is associated with their unique Ethereum Address (EA) on-chain to maintain privacy.

### C. Self-Sovereign Identity (SSI)

Instead of servers, such as those in typical centralized identity management systems (IdM), in SSI, the users access their wallets through their Dapps and control access to their sensitive information. Therefore, the users of a system are empowered and have the freedom of controlling their identity and credentials [19], [20]. In conventional identity management systems, organization resources are restricted to authorized individuals. The Open Authentication (OAuth), and the OpenID Connect are examples of the traditional IdM systems.

An effective identity system would have an identity provider, a service provider, and users. Identity providers provide authentication, registration, and identity-related services to users and service providers. An identity provider can be a third-party service independent from the service providers. The service provider typically requests the identity provider for validation and authentication of the identity claimed by a user [21].

Sovrin, uPort, and ShoCard are three of the blockchain-based identity management systems that work on manifesting a digital identity without relying on a centralized service [22]. A key feature of blockchain-based identity management systems is that it relies on peer nodes for the storage of identity information instead of a central server. Moreover, they should maintain authentication, trust, and privacy. Some proposed blockchain-based identity systems keep the users anonymous and rely on an attribute reputation model in addition to an SSI system. The effectiveness of the blockchain-based solutions depends on having a large community where users would request attestations and certificates to be able to verify the proof of identity and individuality.

There are many proposed blockchain-based identity management systems and they have several strengths and weaknesses. Some of the strengths of the proposed systems include zero-knowledge protocol, zero trust model, universal discoverability, selective anonymity, data transparency, and immutability. The cost of the infrastructure, as well as key management, are major drawbacks that need to be considered when choosing an SSI system. A gradual upgrade of existing identity management systems can help in reducing the cost burden. In password-based systems, a lost or forgotten password can be easily reset. However, in blockchain-based SSI systems, losing the private key leads to an asset loss [23].

Table 1 summarizes the strengths and weaknesses of a decentralized and centralized identity management system. In our implementation of a blockchain based solution for the creation of digital health passports and immunity certificates, we have chosen a decentralized SSI management system. In a decentralized identity management system, the storage is distributed and the users’ freedom is maintained with no risk of identity abuse. Self sovereign identity can be used in digital health passports and immunity certificates as it offers security and transparency making it a compelling tool. Also, using SSI provides the users with the opportunity to share information as much as necessary when needed only. Therefore, the users’ freedom is respected and fulfilled.

**TABLE 1:** Comparison Between Centralized and Decentralized Identity Management Systems

Feature	Conventional Identity Management Systems	Blockchain-based Identity Management Systems
Governance	Centralized	Decentralized
Freedom	Users risk identity abuse	Users regain control over their identity
Storage	Centralized Servers	Distributed across nodes
Key Management	Losing a password can be fixed by resetting the password	A lost private key means a loss of the assets
Identity Change	Can be easily achieved by changing information stored in the servers	Identity can be changed but it is more cumbersome as it is under the control of the user

Personally identifiable information (PII), medical records, and immunity certificates are under the control of the user in Self Sovereign Identity (SSI). SSI holders should be able to hold the access rights to their credentials including PII. In our system, test-takers, patients, and on-chain participants should all deal with a decentralized SSI management system instead of relying on a third party or a centralized system. Managing and updating their credentials (PII, immunity, medical records, etc.) are in their control with the ability to choose what is kept private. A centralized database poses a high risk of a breach that may end up compromising the security of the data. For instance, 140 million people were affected by the Equifax breach [24]. A decentralized SSI that does not depend on centralized databases can help in alleviating data breach risks and eliminating identity abuse issues.

### D. Blockchain Client: Staking Gateways

Blockchain clients are an essential part of the proposed solution architecture. They are required to bridge the communication between the blockchain network and the event listeners. Depending on the amount of information they need to hold, such as block headers or transaction information, the storage capacity, RAM, and bandwidth is chosen. There are full nodes, light nodes, and super light nodes based on the chosen requirements.

The blockchain client can be an Infura, Linxa, Meatmask, or Geth gateway. Those clients if used as the only source of information from the blockchain network to the listeners will turn the solution into a centralized one. They do not add any security features to the blockchain network. Their only objective is to handle the data to the intended listeners in a tamper-proof manner.

Therefore, a better solution is to use staking gateways. Multiple blockchain clients are used together to adequately carry the information from the blockchain network to the requesting parties. This adds a level of security and decentralization to the solution. SlockIt and Chainsafe are two examples that depend on hybrid clients where a misbehaving gateway is penalized and might risk losing its stake. The suspecting gateways can submit a proof of fraud to the blockchain network and a penalty is imposed on the malicious client.

Furthermore, blockchain gateways are collaborating to build a distributed web and avoid decentralization. Cloudflare and Infura are two blockchain gateways that are cooperating together to build a decentralized web gateway. Caching, load balancing, and Argo Tunneling are used to boost the performance and securely deliver the content.

### E. IPFS Data Confidentiality

IPFS is used for off-chain documents stored in a decentralized way. The documents related to COVID-19 testing, identification, and travel would be too expensive to store on-chain. Therefore, storing this content in a decentralized and secure way is mandatory. IPFS storage is distributed and public to everyone. Consequently, the information stored on IPFS should be encrypted and only authorized entities should be able to read the plaintext content.

Therefore, in our system design, the data owner encrypts the files that are uploaded to the IPFS servers using a symmetric key. Additionally, our system allows multiple people to access the content on the servers while maintaining confidentiality [25], [26]. Those entities include hospitals, testing centers, airport authorities, airline agents, employers, and academic officers. Hence, a mechanism that allows the content to be shared based on the permission of the data owner should be applied. Also, the system should allow the only authorized receiver to access the clear content.

Hence, we designed our solution with proxy re-encryption schemes to enable multiple parties to access the IPFS content confidentially [27]. Furthermore, this also allows our solution to be distributed with the availability of multiple trusted oracles. First, the data owner encrypts the content using a symmetric key. The encrypted information is uploaded to IPFS and only its hash is stored in the smart contracts. Only the data owner knows the symmetric key used to encrypt the uploaded information.

Second, in simple terms, the oracles need to recreate a different key for each receiver without knowing the original key. Hence, the oracles have a copy of the encrypted symmetric key. The data owner encrypts the symmetric key with their public key and sends it to the oracles. When a receiver requests the information, the proxy server would send the request to the data owner. The data owner creates a new key using their private key and the receiver’s public key. The key is sent to the proxy for re-encryption. After the re-encryption process, the new key created is sent to the data receiver. The data receiver then decrypts the key using their private key to get the original symmetric key. The receiver finally uses the symmetric key to decrypt the content on IPFS.

**FIGURE 2.**
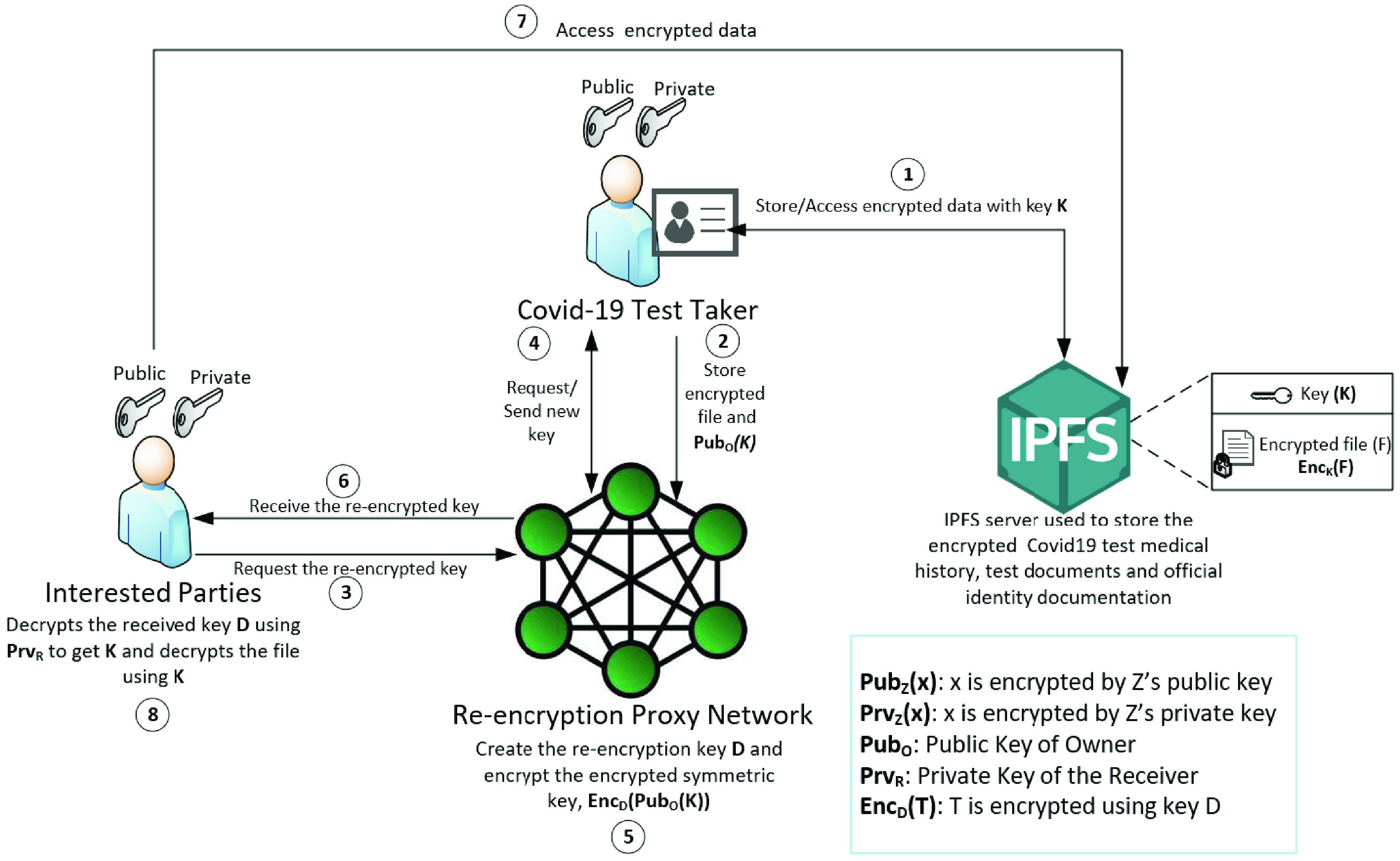
Re-encryption proxy scheme process details.

Figure 2 shows the detailed steps involved in proxy re-encryption. The COVID-19 test-taker uses a symmetric key to encrypt the medical records and PII. Subsequently, the encrypted data is stored on IPFS along with the symmetric key. The hash of the encrypted information is only stored on-chain in the smart contract. Using this way, only the data owner knows the symmetric key as it is without encryption. Once a receiver requires to retrieve the data stored on IPFS, the data owner recreates a new key using the receiver’s public key and its own private key. The newly created key is sent to the proxy network for re-encryption.

As illustrated in the diagram, the COVID-19 test-taker stores the encrypted file, $Enc_{K}(F)$ and the key, $K$ on IPFS. When any of the interested parties (academic institutions, travel agents, and transportation facilities) need access to the IPFS file content, they communicate with the re-encryption proxy network. network. The COVID-19 test-taker would then send a key to the re-encryption proxies to create a new key, $D$ which can be used by the interested parties as earlier.

Consequently, the receiver can also compute the hash of the content and compare it to the hash stored in the smart contract, thereby ensuring data integrity and immutability.

### F. Interested Stakeholders

Several listeners can gain benefit immensely from the events generated by the on-chain participating entities. For example, airline agents, airports, employers, academic facilities, and public transportation systems.

Since COVID-19 is highly contagious, it is important for all the sectors where human interaction is inevitable to ensure protection against COVID-19. Therein, the proposed solution will enable them to consult the on-chain records and events to identify anyone using their utilities or is a potential user of their services.

Listeners are on-chain interested entities that interact with the blockchain client to receive the filtered events from the public blockchain. Events are transparent and accessible to everyone on-chain. It is part of the intrinsic features of blockchain that reflect its transparency, trust, and immutability characteristics. Hence, no management system is required. Events in our solution carry information about test-takers and affiliated testing centers. Therefore, if a listener would require access to the identification documents of a patient, they can use the IPFS hash in the patient’s smart contract to access the required information.

Any documents stored off-chain that might be required for validation, such as the COVID-19 test results, COVID-19 test date, medical history, travel history, valid passport, and identification documents can be accessed through IPFS. The IPFS hashes are stored in the smart contracts and can be accessed by the listeners and participating entities.

## III. Implementation Details

In this section, we present the implementation details of the proposed system. The code is written using the Remix IDE [28] which is used for compiling and testing the smart contracts. We have developed four smart contracts; namely, MoFA, MoH, COVID-19 Testing Center, and the Patient smart contract.

**FIGURE 3.**
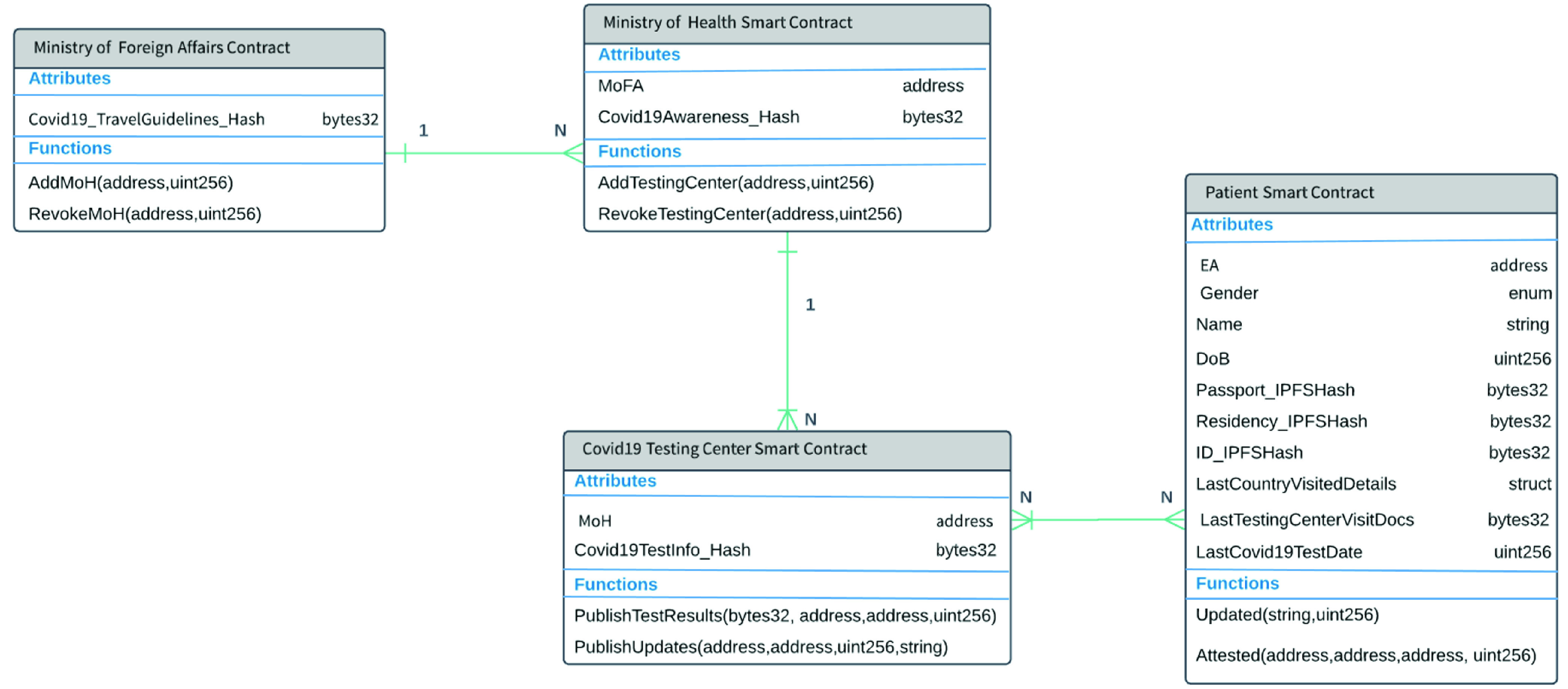
Entity Relationship diagram showing the different interactions between the smart contracts.

Figure 3 shows the entity-relationship diagram and describes the functions in each smart contract along with the attributes. As can be seen in this figure, there are four types of smart contracts. The MoFA smart contract can be associated with several MoH smart contracts. The MoH smart contract can point to multiple COVID-19 testing center smart contracts. The patient smart contract can also be associated with one or more testing center smart contracts. In our design, any documents are stored on IPFS. Therefore, the hash of types bytes32 is stored as an attribute in the smart contracts as can be seen in figure 3. The main functions of the smart contracts rely on generating events to notify all listeners of the actions taking place. This also reduces the on-chain cost and makes use of the available immutable logs of the blockchain. Further details of each algorithm are presented below.

### 1) MoFA: Country Addition

A country can be added to the approved list of affiliated countries using algorithm 1. In this algorithm, the owner of the MoFA contract is the only authorized entity that can add to the approved countries. This function would then generate an event to all the participating entities to notify them about the update along with the time. The blockchain client (BC) and gateway will use this event as part of its filtered events when communicating with the interested entities so they update their records accordingly.

**Algorithm 1:**
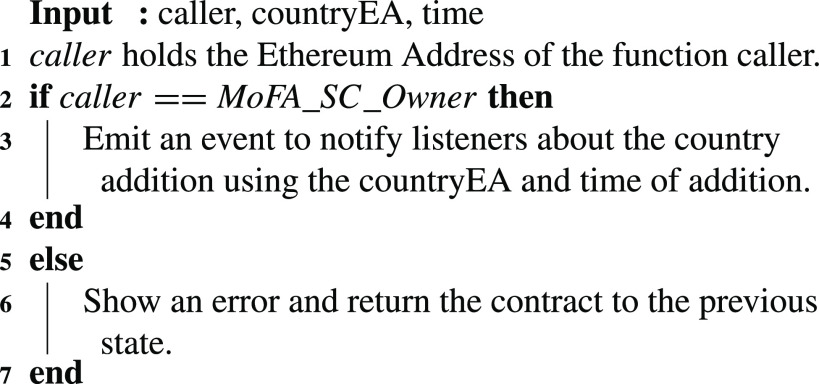
MoFA: Country Addition

### 2) MoFA: Country Withdrawal

A country that should be withdrawn based on the MoFA rules and regulations is revoked using the algorithm 2. In this algorithm, the owner of the MoFA contract is the only authorized entity that can revoke a previously added country. This function would then generate an event that is logged for the participating entities to be notified about the update and its effective time. The blockchain client (BC) and gateway will use this event as part of its filtered events to notify interested parties about the revoked country.

**Algorithm 2:**
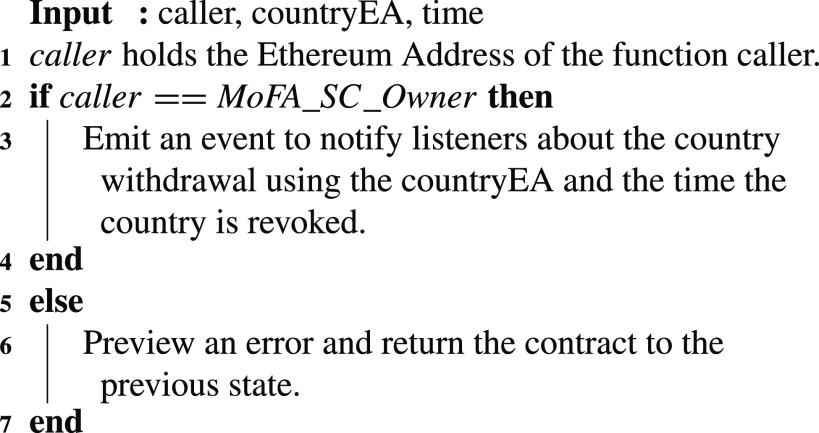
MoFA: Country Withdrawal

### 3) MoH: Testing Center Addition

A COVID-19 testing center that meets the criteria set by a country’s MoH is added to the affiliated testing centers of the MoH. The owner of the MoH smart contract is the only authorized entity to execute the function call that adds the approved testing center. This function generates an event as described in algorithm 3. The event shows the time the update is effective as well as the testing center EA.

**Algorithm 3:**
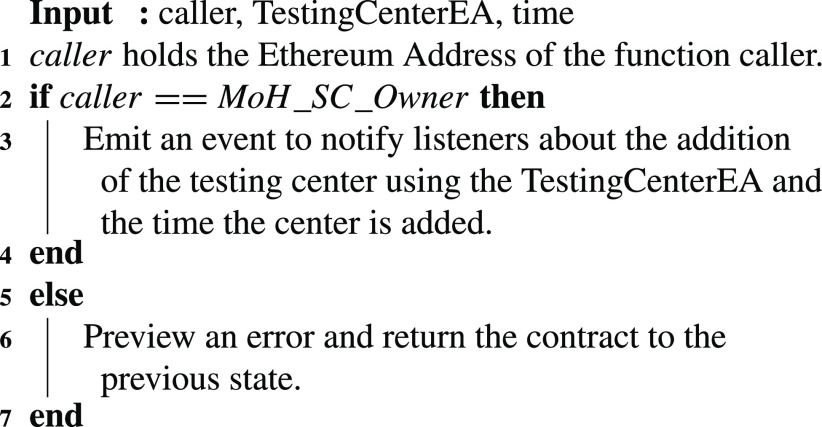
MoH: Testing Center Addition

### 4) MoH: Testing Center Withdrawal

A COVID-19 testing center that needs to be withdrawn from the list of previously approved testing centers can be revoked through algorithm 4. In this function, the time the revoked testing center is withdrawn is announced as an event which is communicated to all interested entities.

**Algorithm 4:**
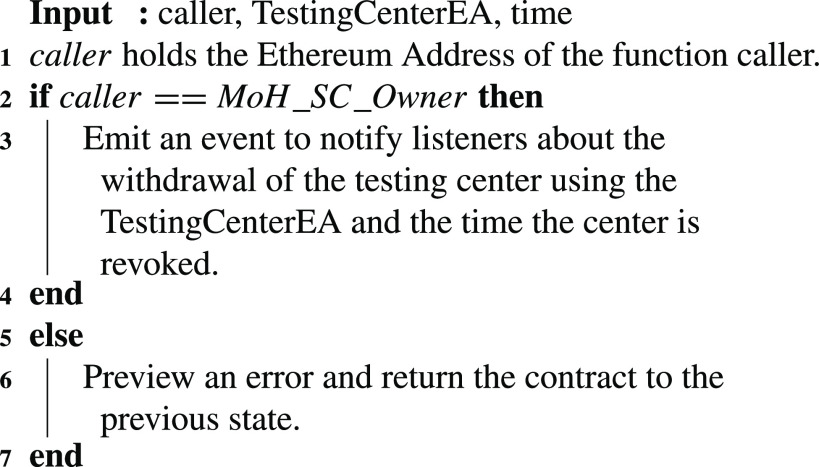
MoH: Testing Center Withdrawal

### 5) COVID-19 Testing Center: Test Results Completion

The COVID-19 testing center smart contract announces the results of the COVID-19 test using the algorithm 5. The algorithm shows how an event is created once the results are out. To maintain the privacy of the patient, only the IPFS hash of the results is logged as well as the patient’s EA and patient’s smart contract EA. The time when the result is broadcast is also part of the notification. This event can then be filtered by the interested entities and the IPFS hash can be used to verify the results of the test.

**Algorithm 5:**
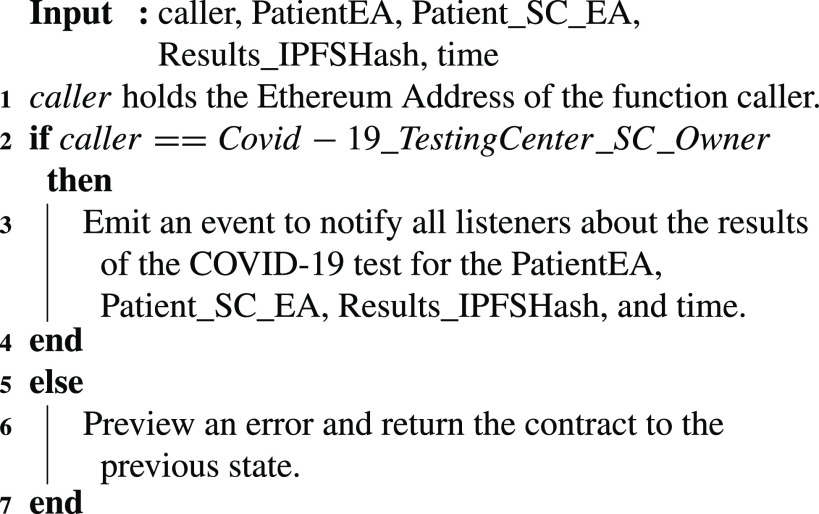
COVID-19_TestingCenter: Test Results Completion

### 6) COVID-19 Testing Center: Patient Updates

Using algorithm 6, a COVID-19 testing center can publish updates in the form of events to notify the participating entities of any news. The announcement is in the form of an event that has the message as well as the time and the patient’s EA and smart contract address. This announcement can be about any update any update related to the patient. For instance, it can be regarding the expected time a COVID-19 test result will be available or a transfer of a patient’s file from one testing center to another.

**Algorithm 6:**
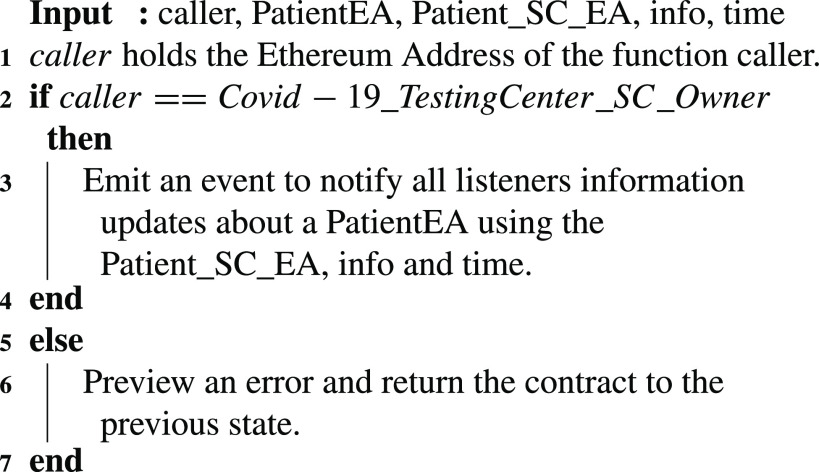
COVID-19_TestingCenter: Patient Updates

### 7) Patient: Patient SC Updated

The patient smart contract has detailed information about a patient, such as the passport documents, valid identity, and other information that needs to be always up to date. Therefore, a patient can update the information stored on IPFS as well as in the smart contract, and execute the update function as shown in algorithm 7. This function generates an event that shows the time an update took place. This function can only be executed by the patient. The updated information is updated in person at the Testing Center just like the registration as well as by the patient through IPFS and onchain through the immutable events. The owner of the Testing Center smart contract would then execute algorithm 8 to perform the attestation.

**Algorithm 7:**
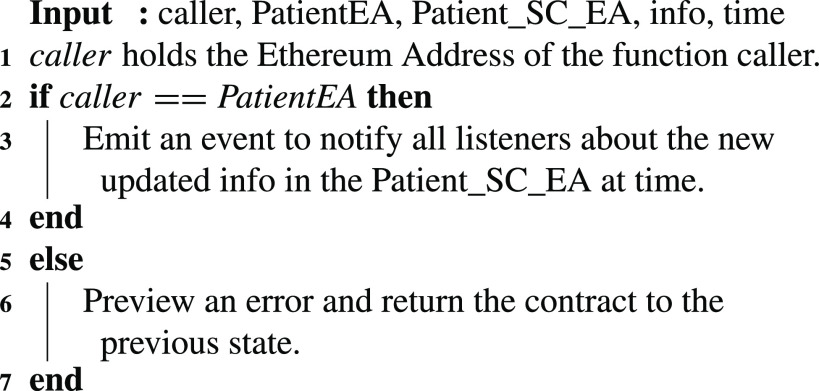
Patient: Patient SC Updated

Algorithm 8: **Patient: Patient SC Attestation**
**Input**: caller, PatientEA, Patient_SC_EA, TestingCenterEA, time
1 *caller* holds the Ethereum Address of the function caller.
2 Emit an event to notify all listeners about the attestation of the Patient_SC_EA by the caller using the TestingCenterEA, PatientEA and the attestation time.

### 8) Patient: Patient SC Attestation

Once a patient smart contract is updated, it must be attested by an affiliated testing center from the MoH. The attestation function as shown in algorithm 8 should be executed by the owner of the COVID-19 testing center smart contracts. The event generated as a result has the testing center EA, the patient EA as well as the patient’s smart contract EA. It also contains the time of the attestation. This attestation is important to notify the listeners that the update is legitimate and approved by an authorized entity. The application that listens to this event checks the EA of the entity that performed the attestation to ensure it is an approved and legitimate EA. An approach that involves an external call from the Testing Center smart contract itself would cost a lot as compared to the implemented way, where the current cost is negligible.

## IV. Testing and Validation

This section describes the testing and validation of the four smart contract functions which form the core of the proposed solution. Each smart contract has modifiers and function calls that were tested to ensure that only the intended and legitimate Ethereum address holder can execute the functions. Moreover, events in the logs are also verified to ensure the correct flow of information and data. The Ethereum address of the owners of the four smart contracts are presented in table 2.

**TABLE 2:** Ethereum Addresses for Smart Contract Owners

Smart contract	Ethereum address
Ministry of Foreign Affairs	$0\times 14723$A09ACff6D2A60DcdF7aA4AFf308FDDC160C
Ministry of Health	$0\times 4$B0897b0513fdC7C541B6d9D7E929C4e5364D2dB
Patient’s smart contract	0xdD870fA1b7C4700F2BD7f44238821C26f7392148
COVID-19 testing centre	$0\times 583031$D1113aD414F02576BD6afaBfb302140225

The rest of this section shows the execution results of the function calls and their outputs using the Remix IDE [28]. All the functions in the smart contracts can only be executed by the authorized parties. This is ensured using modifiers which restrict the execution to a certain Ethereum address only. Moreover, each smart contract with its associated functions is presented below and a screenshot showcasing the produced output. The screenshot shows the name of the function that was successfully executed in the remix environment as well as the address of the caller, the event name, logs, and the associated event arguments.

### A. MoFA: Country Addition

**FIGURE 4.**
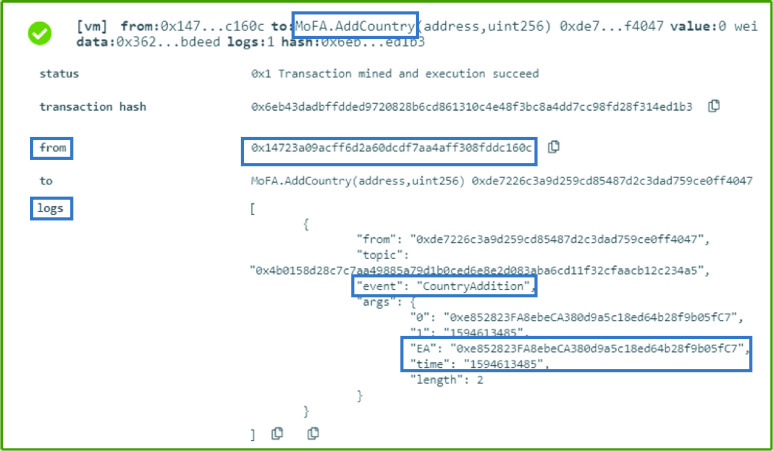
Logs showing a successful country addition.

The MoFA can add a country to its list of affiliated countries using a function called $AddCountry$. This function creates an event announcing the affiliation of anew country’s MoH to all listeners. The affiliated MoH department’s Ethereum address is used as an input to the function as well as an output in the event. Moreover, the time the affiliation took place is also part of the event as presented in figure 4.

### B. MoFA: Country Withdrawal

**FIGURE 5.**
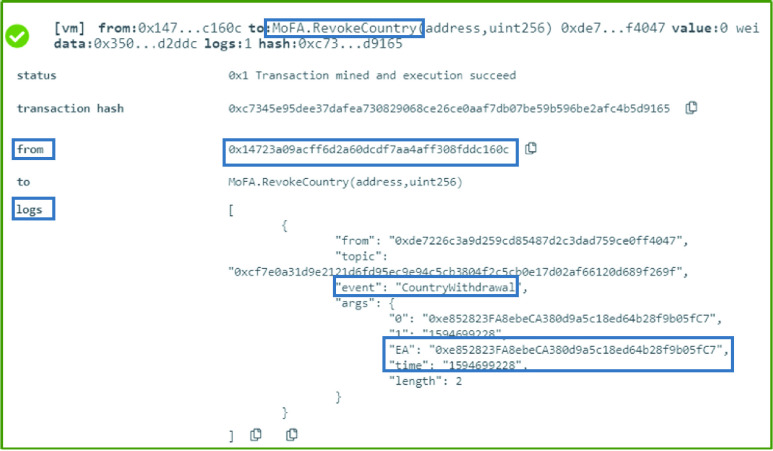
Logs of a country successfully revoked by the MoFA.

The MoFA has the right to revoke any previously affiliated country. Therefore, this is done through the execution of the function $RevokeCountry$, as can be seen in figure 5. The country is withdrawn through an event that announces to all the interested parties that the country with the specified Ethereum address is no longer affiliated with the MoFA. The event also includes time information which shows the date and time the decision is effective from as presented in figure 5.

### C. MoH: Testing Center Addition

**FIGURE 6.**
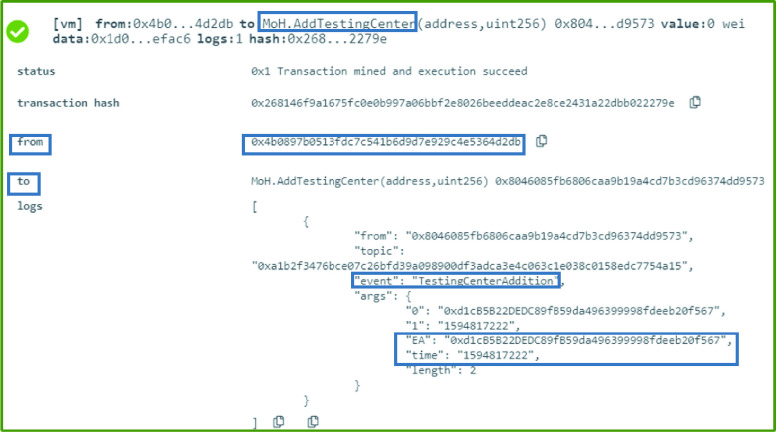
Logs a successful addition of a testing center by the MoH.

The owner of the MoH smart contract is the authorized entity that can execute the $AddTestingCenter$ function. The testing center that is affiliated with the MoH is added using the testing center’s Ethereum address and the date the new decision is effective. This is announced through an event to all the interested listeners. The function was executed successfully and the event contains the EA as well as the time in uint256 as illustrated in figure 6.

### D. MoH: Testing Center Withdrawal

**FIGURE 7.**
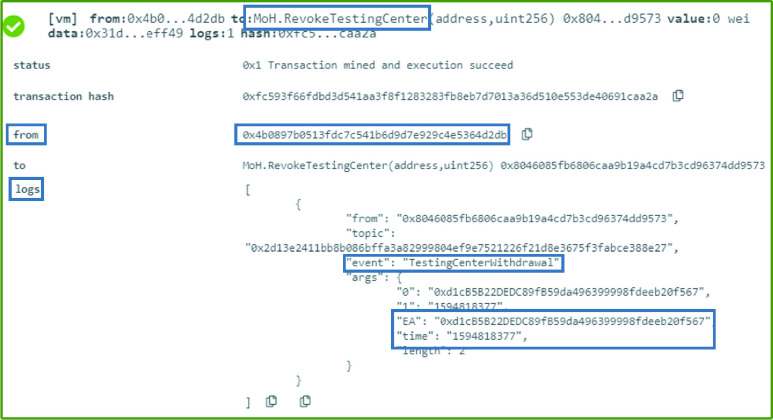
Logs showing an event generated announcing the withdrawal of a testing center by the MoH.

A testing center that no longer complies with the MoH’s regulation and requirements can be revoked from the authorized list. This can be achieved by executing the function $RevokeTestingCenter$ which generates an event that shows the revoked testing center’s Ethereum address as well as the date and time the new decision is effective from as illustrated in figure 7.

### E. COVID-19 Testing Center: Test Results Completion

**FIGURE 8.**
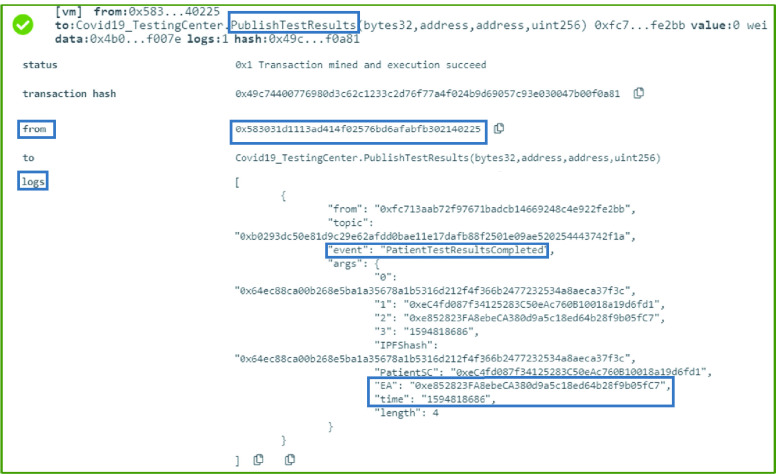
Logs showing an announcement with the test results IPFS hash by the COVID-19 testing center.

Once the results of the COVID-19 lab test are out and ready for collection, the function $PublishTestResults$ is executed. This function is successfully executed executed where an event is generated as a notification to all interested participating entities. Moreover, the event includes the COVID-19 test-taker’s Ethereum address, smart contract address, the time the event is published, and the IPFS hash of the test results. The logs and event details are demonstrated in figure 8.

### F. COVID-19 Testing Center: Patient Updates

**FIGURE 9.**
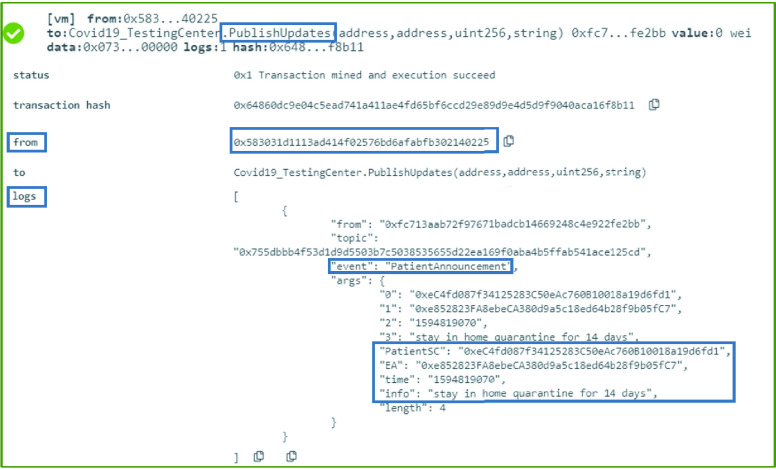
Logs showing a successful announcement by the COVID-19 testing center.

The COVID-19 Testing Center can publish updates on any of its patients. Therefore, the function $PublishUpdates$ was tested successfully to verify that the event created as a notification of an announcement contains all required details. Those details include the patient’s smart contract address, the time of the announcement, the information that needs to be published as well as the Ethereum address of the patient as shown in figure 9.

### G. Patient SC: Patient SC Updated

**FIGURE 10.**
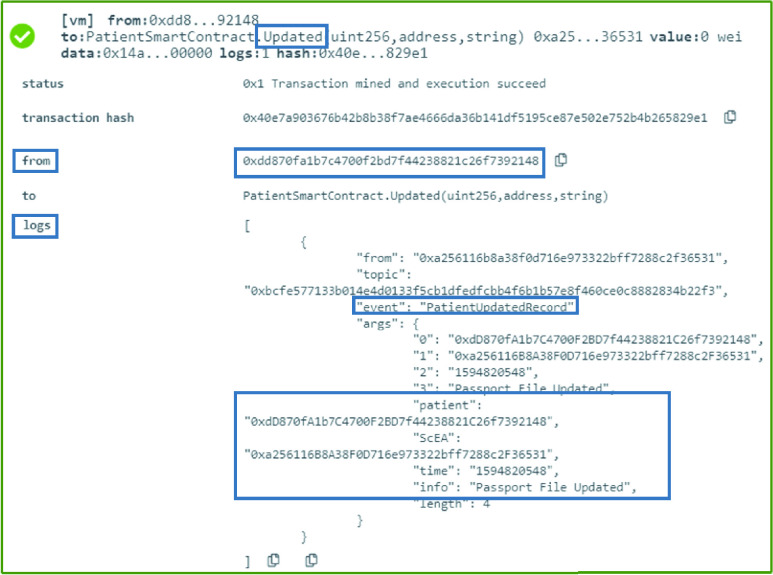
Logs indicating an update occurred to the patient’s SC data.

A patient smart contract can be updated by its owner. Therefore, any new update has to be attested and verified. The owner of the smart contract uses this function every time an update is announced using an event about the change. An event is generated as can be seen in figure 10. The event shows the patient’s Ethereum address as well as the smart contract address, the time the event was created, and information about the new update. All the information about the event as well as the function are logged and can be verified in the immutable logs as illustrated in figure 10.

### H. Patient: Patient SC Attestation

**FIGURE 11.**
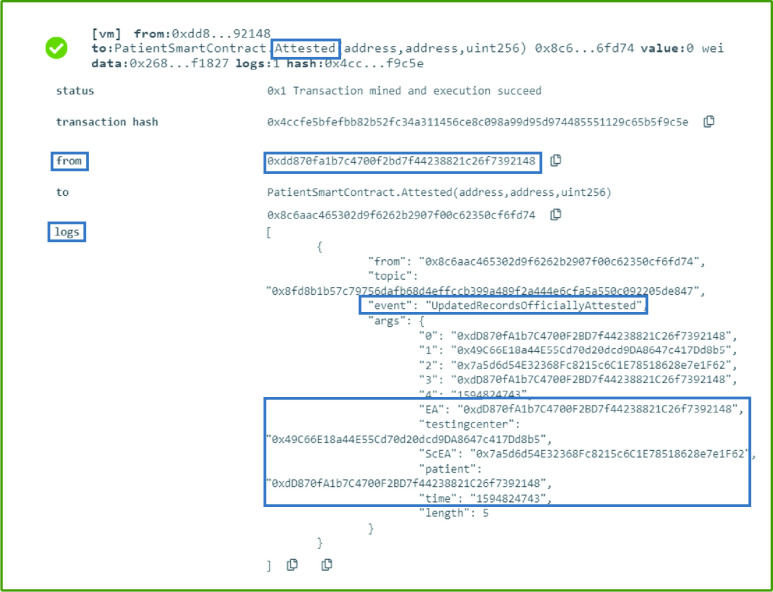
Logs showing a successful attestation of the patient SC by an authorized testing center from the MoH.

Any update in the patient smart contract should be attested by the COVID-19 testing center to be a legitimate update. Therefore, the owner of the COVID-19 testing COVID-19 testing center associated with the patient executes this function call to generate an event as shown in figure 11. The event informs all participating entities the time of the attestation, along with the patient’s Ethereum address and smart contract address. It also includes the Ethereum address of the caller and the COVID-19 testing center’s Ethereum address.

## V. Discussion

In this section, we evaluate our proposed solution with respect to security and financial cost to assess its feasibility for use within a real-world setting.

### A. Security Analysis

Blockchain networks are highly secure, resilient, and robust as they are based on cryptographic fundamentals which underpin them. However, smart contracts, if not carefully written, can be easily exploited. Smart contracts should be checked for reentrancy errors, infinite loops, and other common forms of software bugs which can make the smart contract highly vulnerable for hackers. Therefore, we have used the smart contract checking tool SmartCheck [29] which assesses the code against several bugs and vulnerabilities. Those bugs include reentrancy, Denial of Service (DoS) by an external contract, costly loops, private modifiers, integer division, malicious libraries, and locked transfers. The SmartCheck tool provides a *no bug* report at the end of a successful security check. Our smart contracts code has passed the check of the SmartCheck successfully which gives us confidence in its resilience against known bugs and vulnerabilities.

Blockchain incorporates intrinsic security features that make it ideal for many applications. It facilitates developing trusted and secure solutions that are resilient and tamper-proof. Moreover, blockchain helps in achieving several security characteristics, such as integrity, availability, authorization, non-repudiation, and confidentiality. In this section, we will highlight the importance of each security requirement and explain how blockchain has paved the way for its applications to gain benefit from them.

**Integrity** is one of the fundamental security requirements that is critical to the benign function of applications. Due to its cryptographic fundamentals, blockchain has built-in integrity protection as it is a tamper-proof and immutable ledger. All the information that is stored in the smart contracts and that is part of the logs can not be amended or deleted. Its complete history since its creation is preserved including all its transactions. Therefore, data integrity is well-maintained using the resilient blockchain network supported by its underpinning cryptographic foundations.

**Availability** is another security requirement that is facilitated by the blockchain network. The blockchain network and smart contracts are always available for their participating entities. Transactions can take place at any time in a robust and secure way. This is enhanced further through the decentralized function of a blockchain network which protects against the single point of failure.

**Authorization** is a vital security feature in any application. For example, in our proposed solution, it is important that only COVID-19 testing centers publish updates about the test-takers as well as their results. Therefore, modifiers are used to restrict transactions from being executed if any other illegitimate Ethereum address tries to make a function call. Hence, all functions are restricted based on the Ethereum address of the authorized parties only.

Moreover, **Non-repudiation** is a security feature that ensures a participating entity cannot deny its actions. All transactions that take place on-chain are logged with their details and the Ethereum address of the caller, which are stored in a tamper-proof manner. Therefore, the entity that makes the function call cannot deny that it has executed a transaction as everything is part of the immutable logs.

In addition to the above, blockchain also offers **confidentiality and privacy**. Ethereum is a public network that makes all transactions available to the public. However, based on the application requirements and the scenario context that the application fits in, a solution can be developed in a permissioned blockchain network. Hyperledger Fabric as well as Hyperledger Besu are two permissioned networks that offer confidentiality and privacy. Membership Service Provider (MSP), channels, and groups are ways that those permissioned networks use to capture identities and engage only the needed participating entities together. Therefore, only the authorized entities can communicate together privately.

**Man-in-the-Middle (MITM) Attack** is a well-known vulnerability that is especially exploited by intruders through covert channels such as *backdoors*. Malicious attempts aiming to achieve MITM attacks intercept messages to take advantage of vulnerable authentication protocols. In a typical MITM attack, an unauthorized intruder tries to capture the messages between two parties by impersonating the victim. This is dangerous as messages will no longer be private and can also be manipulated by the intruders before re-sending. Other issues that can take place due to a MITM attack include Domain Name System (DNS) spoofing, and session hijacking.

On the other hand, any communication on-chain is secured against MITM attacks as messages are digitally signed by the senders and the Ethereum Address (EA) is always part of the sent signed transaction. A message $X$ sent by $Z$ is concatenated with the public key, $X\|Pub_{Z}$. Then the whole message is digitally signed using the private key, $Prv_{Z}(X)\|Pub_{Z}$. Furthermore, the receivers can easily check the authenticity of the sender by computing the hash of the public key, $H(Pub_{Z})$ and verifying it with the EA sent as part of the privately signed message, $X$. The hash of the public key should give the EA of the sender and should match the EA sent in the message [30]. The verification of hashed EA assures the receiver that the message indeed is sent by the claimed identity.

**FIGURE 12.**
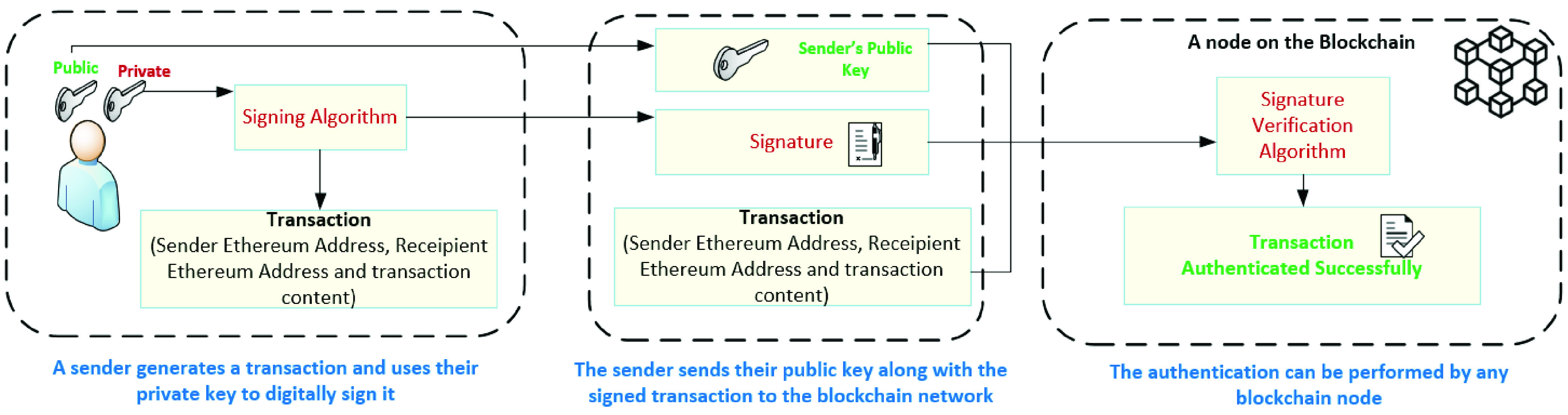
Authentication process of a blockchain transaction.

Figure 12 presents the authentication process of a blockchain transaction for the proposed system. The sender sends their public key along with the signed transaction. The signed transaction is then authenticated by the blockchain nodes using the signature verification algorithm. This authentication process helps in ensuring all transactions on-chain are digitally signed and verified. Moreover, our blockchain-based solution is secure onchain as well as off chain during the communication with the re-encryption proxies. It is vital to maintain a secure communication between all parties on-chain and off chain. Therefore, SSL/TLS X.509 public key certificates are used to associate a public key with an individual or organization. Hence, the public keys are associated with the re-encryption proxies and users. Additionally, the proposed system uses hashes to protect against fake certificates that intruders might create when initiating a communication. For instance, when a user receives a signed message from the re-encryption proxy, the public key, $Pub_{proxy}$ is used by the receiver to validate the message. The receiver decrypts the signed message and computes the hash of the public key, $H(Pub_{proxy})$. Then the user would validate the sender by comparing it to the EA received as part of the signed message.

### B. Cost Analysis

Transactions on the Ethereum blockchain incur a certain transaction fee. The transaction cost as well as the execution cost are always part of the logs in the Remix environment. A unit of gas holds the unit *Gwei* and is paid for in Ether. The miners prioritize transactions that hold a higher amount of *Gwei*. Therefore, the ETH Gas Station [31] provides different speeds for the transactions based on the gas prices offered. It is always necessary to estimate the gas costs while developing a smart contract and so as to eliminate extra charges. Loops, arrays, mappings, variable storage, and manipulation as well as data types play a major role in transaction costs. The feasibility of the solution and efficiency is extremely important. Hence, our solution leverages the blockchain immutable properties and depends on the events and logs rather than on-chain storage.

Gas prices can also vary depending on the date and time. It is worth mentioning that we are aware of the current high gas prices due to the highly congested network. Currently (July 2020) the gas prices are at peak levels which make the transaction fees cost higher than what they usually would cost on normal off-peak days. Consequently, in our analysis, we would like to fairly evaluate our solution. Therefore, we present the costs of the algorithms in USD in off-peak days. We have used the gas prices provided by the ETH Gas Station on May 7, 2020, where the fastest, fast, average, and cheap gas prices were found as 10, 6.6, 4, and 4 Gwei, respectively, [31]. In our cost analysis, we present the cost in USD depending on the average gas price of 4 Gwei as can be seen in table 3.

**TABLE 3:** Gas Cost in USD of the Smart Contract Algorithms

Algorithm	Transaction	Execution	Cost
Number	Gas	Gas	USD
1	25555	2491	0.024533
2	25533	2469	0.024512
3	25533	2469	0.024512
4	25555	2491	0.024533
5	29517	3125	0.028336
6	31915	4627	0.030638
7	29216	4296	0.028047
8	27881	3409	0.026766

Table 3 shows the transaction cost as well as the execution cost in Gwei. The highest transaction cost which yielded to the highest cost of $ 0.030638 can be seen for algorithm number 6. The cost is considered to be low even if it is highest between the algorithms presented in our solution. Algorithm 6 generates an event showing patient updates by the COVID-19 testing center. It has four attributes as part of the event and the information announced by the testing center is a string. Therefore, since it is the only string used in our solution, algorithm 6 has the highest cost. As a result of the algorithms do not contain any loops or arrays, therefore cost is minimal as expected.

### C. Generalization

Our solution tackles an important problem where the travel history and medical history of a COVID-19 test-taker or patient can be traced and tracked easily. Using easily. Using the immutable logs, a patient and a COVID-19 test-taker can safely engage in social activities and institutions. Community managers and stakeholders can verify the immunization records as well as travel history, medical and quarantine information in an efficient and trusted way. This solution leverages the blockchain security features and mitigates the spread of the highly contagious COVID-19 virus.

Furthermore, due to inherent similarities with other infectious diseases, this solution can be easily adapted to other types of viruses or diseases. The fundamental concept underlying the proposed solution is a medical passport that accurately records the symptoms for a type of a disease through the medical information provided by the patient and the medical entity. The medical files and other types of identity information are stored on the IPFS (off-chain storage). Only the hash is stored in the patient’s contract which acts like their medical passport and can therefore be valid for any disease. In addition, for any disease that needs to be traced, the medical testing centers or entities must be affiliated with a relevant authority, such as the MoH. Furthermore, due to the global implications of such infectious diseases, MoFA is a critical stakeholder in any response strategy. Hence, the MoH is envisaged to be affiliated by a higher authority, such as the MoFA within the context of the solution we have proposed in this paper.

Currently, immigration and some travel destinations require a medical examination and flu shots before traveling. This information can also be part of the medical passport of travelers which is easily traced using the blockchain smart contracts and tamper-proof logs. Therefore, our solution can immensely change the way travelers are verified against diseases whether or not a pandemic exists. It is a general skeleton for any type of tracing needed against diseases as well as a profile of test-takers along with their symptoms, travel and medical history, and immunization records.

## VI. Conclusion

This paper presents design, development, and evaluation of a blockchain-based solution for digital health passports with immunity certificates. The proposed system helps in mitigating the spread of infectious diseases in general and the COVID-19 disease in particular. The paper presented four smart contracts that rely on negligible on-chain storage and leverage on-chain events and notifications. In our approach, we incorporated self-sovereign identity, re-encryption proxies, and associated biometric information to the unique Ethereum addresses of the participating entities. We evaluated the algorithms for the proposed solution using a detailed cost analysis check as well as for known vulnerabilities using the *smartcheck* software. We believe that our solution paves the way for efficient solutions that can help in stopping the transmission of infections through accurate and timely recording of events in a tamper-proof manner.
